# Fecal microbiota transplantation repairs intestinal permeability and regulates the expression of 5-HT to influence alcohol-induced depression-like behaviors in C57BL/6J mice

**DOI:** 10.3389/fmicb.2023.1241309

**Published:** 2024-01-05

**Authors:** Dezhi Li, Wei Liang, Wentong Zhang, Zhiqiang Huang, Haipeng Liang, Qing Liu

**Affiliations:** ^1^School of Health Science and Engineering, University of Shanghai for Science and Technology, Shanghai, China; ^2^The Fourth Affiliated Hospital of Guangxi Medical University, Liuzhou, China; ^3^Qingyang City People's Hospital General Surgery, Qingyang, China

**Keywords:** alcohol dependence, fecal microbiota transplantation, 5-HT, brain gut dysfunction, gut microbiota

## Abstract

The epidemic of alcohol abuse affects millions of people worldwide. Relevant evidence supports the notion that the gut microbiota (GM) plays a crucial role in central nervous system (CNS) function, and its composition undergoes changes following alcohol consumption. Therefore, the purpose of this study was to investigate the effect of reconstructing the gut microbiota by fecal microbiota transplantation (FMT) on alcohol dependence. Here, we established an alcohol dependence model with C57BL/6J mice and proved that FMT treatment improved anxiety-like behavior and alcohol-seeking behavior in alcohol-dependent mice. Additionally, we found that the expression of the intestinal intercellular tight junction structure proteins ZO-1 and occludin was significantly increased after FMT. FMT repaired intestinal permeability in alcohol-dependent mice and decreased the levels of lipopolysaccharide (LPS) and proinflammatory factors. Moreover, the serotonin (5-hydroxytryptamine, 5-HT) content was significantly increased in alcohol-dependent mouse intestinal and brain tissues after receiving the fecal microbiome from healthy mice. 16S rRNA sequencing demonstrated that FMT markedly reshaped the composition of the gut microbiota and elicited changes in the intestinal barrier and 5-HT levels. Collectively, our results revealed that FMT has a palliative effect on alcohol dependence and explored the underlying mechanisms, which provides new strategies for the treatment of alcohol dependence.

## Introduction

Alcohol is one of the most commonly addictive substances in the world (Carbia et al., 2021). Long-term drinking can lead to psychological and physiological dependence on alcohol, forming alcohol dependence (Gorini et al., 2014). As a chronic relapsing mental disorder, alcohol dependence is characterized by alcohol seeking and losing control of alcohol consumption (Dina et al., 2006). In addition, a series of withdrawal symptoms such as depression, anxiety and insomnia may occur in alcohol dependence (Retson et al., 2015; Hendricks et al., 2017). It has been proved that environmental factors, psychosocial, neurobiological, genetic factors are associated with the development of alcoholism (Arya et al., 2021; Chatterjee et al., 2021; Czerwinska-Blaszczyk et al., 2021). However, the molecular mechanisms underlying alcohol addiction are still not completely understood. More importantly, the medicine base on existing pathogenic mechanisms of alcohol dependence is not effective for patients, a new strategy to solve this problem is urgently needed (Rathinam and Ezhumalai, 2022).

In recent years, the role of gut microbiota has attracted increasing attention. The gut microbiota can impact the central nervous system through various pathways and contribute to the development of neuropsychiatric disorders (Agirman et al., 2021). Recent studies found alcohol exposure altered the composition of the mice, rat and human gut microbiota (Wang et al., 2018; Kosnicki et al., 2019; Carbia et al., 2023), however the most of these studies focused on the effects on the digestive system and ignoring its effect on the nervous system.

The gut-brain axis is gaining ever more traction in fields investigating nervous system disease. Many studies have shown that alcohol exposure can cause changes in gut microbiota (Robinson et al., 2014), and some probiotic supplements have been found to alleviate alcohol-related liver and intestinal damage (Yao et al., 2022). A study went further and found that the translation of fetal microbiota from alcohol dependence mice to healthy mice can resulte the alcohol withdrawal-induced anxiety behavior. The mechanism may be that FMT affects the synthesis of brain neurotransmitters, but the specific mechanism has not been explored (Xiao et al., 2018). Actually, GM is inextricably linked to depression, which is thought to be one of the causes of substance dependence (Kaelberer et al., 2018; Angoa-Perez and Kuhn, 2021). However, short-chain fatty acid production, tryptophan metabolism and cytokine expression regulation, and immune activation are potential pathways of brain-microbial interaction (Carbia et al., 2021). Reduced anxiety-like behavior and decreased serotonin receptor expression were observed in germ-free mice (Wichers and Maes, 2004). As a major neurotransmitter, 5-hydroxytryptamine (5-HT) can regulate the secretion of opioid peptide, gamma-aminobutyric acid, glutamic acid and other transmitters, and plays a key regulatory role in many aspects of emotion, cognition, substance dependence and so on (Li et al., 2023). More than 95% of body’s 5-HT is produced by the enteric nervous system (ENS) (Mayer, 2011) and some species of bacteria, such as *Enterococcus*, produce 5-HT (Clarke et al., 2014). Another important aspect, alcohol causes direct damage to the intestinal epithelial layer. Subsequently, the bacterial products in gut, such as LPS, enter the bloodstream and cause inflammatory response, eventually leading to abnormal brain nerve activity (Blednov et al., 2011). According to the report, the gut microbiota may be a new target for the treatment of alcohol dependence (Leclercq et al., 2019). However, there is currently insufficient evidence to confirm the effects of FMT on alcohol withdrawal symptoms such as depression.

In this study, our goal is to explore the improvement effect of FMT on alcohol-induced depressive symptoms in mice. Moreover, we delved into the underlying functional mechanism of FMT in addressing brain-gut dysfunction resulting from in systemic inflammatory response and the 5-HT pathway, with a view to uncovering its potential as an anti-alcoholic depression.

## Materials and methods

### Animals

In this study, 6 weeks-old male C57BL/6J mice, weighing 18–22 g, were obtained from Jiesijie (Shanghai, China). All mice were housed in a room under a 12 h regular light/dark cycle at an ambient temperature 21°C. The animal studies were approved by the Ethics Committee of China and conducted in accordance with ethical standards.

### The establishment of alcohol addiction model

Twenty-five male C57BL/6 mice, aged 6–8 weeks, were fed with water solution and alcohol solution in double bottles. The concentration of alcohol solution was 3%, 6%, and 10% (v/v), and the concentration gradient of alcohol was increased every 4 days to train the alcohol adaptation ability of mice. After 8 days, the alcohol solution concentration was increased to 10% and feeding was continued for another 16 days. After the end of the addiction cycle, the alcohol solution was withdrawn for 24 h, that is, alcohol withdrawal. Five mice in the control group drank water solution, and the other conditions were the same as those in the experimental group. Change the position of the water bottle every day to avoid position preference.

### Donor stool preparation and administration

The donor mice (control group, *n* = 5) was placed in a sterile stool collection box, and immediately after the stool was discharged, the feces were collected with sterilized forceps into a sterile centrifuge tube in an ice box at 4°C. The donor feces were weighed and immersed in sterile PBS for 15 min. The feces were fully shaken into suspension by oscillator, centrifuged at 800 r/min for 5 min at 4 ° C. Transplantation was performed with the supernatant.

### Open-field test

Open field test (OFT) is commonly used to measure anxiety-like behavior and motor activity in mice (Resmim et al., 2023). Due to the fear of the open field, mice move less in the central area and mainly move in the peripheral area. However, the exploration characteristics of mice lead to their motivation to move in the central area. The reduction of movement time and distance in the central area indicates that the anxiety of mice increases, which can be observed. The mice were placed in an open area (50 cm × 50 cm × 40 cm, length × width × height) for 1 min to acclimate to the environment and then freely explore for 5 min. A video camera was placed above the center of the open field chamber, and the movement of each mouse was recorded by an automatic video tracking system, which was converted by the video analysis system of animal behavior into the distance and time of movement in the central area, the total distance of movement, and the speed, etc., which were used as dependent variables. During the test, 75% ethanol was used to clean the open field for each new mouse to avoid residual odor information affecting the results of the next mouse. Each mouse was tested three times, the movement time and movement distance were averaged.

### Light-dark transition test

The light-dark transition test (LDT) is also a behavioral test to measure anxiety-like behaviors in mice (Rosa-Goncalves et al., 2022). The two sides of the light and dark chambers were equal sized compartments (20 cm × 20 cm × 25 cm, length × width × height), one half of which was light and the other half was dark, and the middle was connected by small holes to allow the mice to walk freely. Rodents have a propensity for darkening and spontaneous exploration of new environments, and the exploratory nature of mice drives them to attempt open-box activity. However, anxious mice quickly moved to the dark chamber in response to the bright light stimulus and rarely left the dark chamber. Therefore, an increase in the time spent in the dark box indicates an increase in anxiety, which can be used to observe the anxiety of the mice. During the experiment, the mice were placed in an open box, acclimated to the environment for 1 min, and then explored freely for 5 min. A video system was installed above the light and dark box, and the retention time and activity of mice in the light and dark box were observed and recorded by an automatic video tracking system. During the test, 75% ethanol was used to clean the open field for each new mouse to avoid residual odor information affecting the results of the next mouse. Each mouse was tested three times, the time spent in the dark box was averaged.

### Enzyme-linked immunosorbent assays

According to the manufacture’s instructions (Elabscience, Wuhan, China), the concentration of 5-HT, LPS, FABP2, TNF-α, IL-4 and IL-18 were determined by ELISA assays. A triplicate of each sample (100 μL) was added to enzyme-linked immunosorbent assays (ELISA) plates. The working curve was established using a reference standard, reaction reagents are added sequentially according to the instructions. Inflammatory cytokine levels were determined by 450 nm absorbance measurements on a microplate reader. Assays were performed in triplicate with three independent experiments.

### Immunohistochemistry

Mice were deeply anesthetized with pentobarbital sodium (40 mg/kg, i.p.), and subsequently perfused with 0.1 M PBS (pH 7.4, 37°C), followed by fixation in 4% (w/v) paraformaldehyde in 0.1 M PBS. The colon and brain tissue samples were sliced into 5 μm sections using a crystal microtome (Leica RM 2135, Wetzlar, Germany), followed by xylene dewaxing and gradient ethanol hydration as described previously. Tissues on slides were equilibrated in 0.1 M Tris-buffered saline solution for a duration of 10 min and blocked with 10% goat serum for 1 h. Subsequently, tissues of hippocampus were incubated with primary antibodies for 1 h, including anti-5HT1A (1:100, ab85615, abcam), or anti-5HT2A (1:100, ab66049, abcam), and tissues of colon were incubated with primary antibody anti-ZO-1 (1:100, ab216880, abcam) or anti-occludin (1:100, ab216327, abcam) for 1 h, respectively. Following primary antibody incubation, the slides were thoroughly washed with PBS and subsequently subjected to secondary antibody incubation. Finally, the slides were mounted with glycerin-based mounting media and examined using a fluorescence microscope.

### RNA isolation and qRT-PCR

Total RNA was extracted from mouse tissues using Trizol (Invitrogen, Carlsbad, CA, United States). The cDNA was produced by using HiScript II QRT Supermix (Vazyme Biotech), according to the manufacturer’s protocol. The mRNA transcription levels were measured according to the instructions of One Step qRT-PCR SYBR Green kit (Vazyme Biotech). The gene β-Actin was chosen as an internal control, and the 2^−ΔΔCt^ method was used to calculate the mRNA relative expression level. Four repeats were taken independently to determine significance. The primers used are listed in Table 1.

**Table 1:** Primers used in this study.

Primer	Sequence (5′ to 3′)	Target gene
5-HT1A-F	TCACCTGCGACCTGTTTATC	5-HT1A
5-HT1A-R	GCTCCCTTCTTTTCCACCTT	
5-HT2A-F	GCTCTTTTCTACGGCATCCATC	5-HT2A
5-HT2A-R	AGTTCTTTTTCTGTCCCACCTG	
5-HT1B-F	GCGATTCCAGACGATTTGGC	5-HT1B
5-HT1B-R	TGCCAGCCATTTTGCTCAAC	
5-HT3-F	CGCACCCCCTTCTTGTAGTT	5-HT3
5-HT3-R	AAGTGCTCTCCATTCCAGGC	
5-HT6-F	TCACAGACTGAACCTCAACCC	5-HT6
5-HT6-R	GGACTTGGTCTTAGTGCCTCA	
ZO-1-F	CCGAGCGAGAATGCGGGGC	ZO-1
ZO-1-R	CGAGGGCAGGGCGGGGGCT	
occludin-F	AAGAGCCTCTGCGCTGAAAT	Occludin
occludin-R	GTGGCAGTCAGACTAGCAGTT	
β-Actin-F	GCTGTCCCTGTATGCCTC	β-Actin
β-Actin-R	AGATGTCAGCGCACCAC	

### 16S rRNA gene sequencing

According to the instructions of the E.Z.N.A Stool DNA kit (Omega Bio-tek, Norcross, GA, United States), microbial DNA was extracted from the stool. Next, the purity and concentration of the DNA extraction were determined by a NanoDrop 2000 spectrophotometer, and the quality of the extracted DNA was measured by 1% agarose gel electrophoresis. The V3–V4 region of 16S rDNA gene was selected for PCR using 338F (5′-ACTCCTACGGGAGGCAGCA-3′) and 806R (5′-GGACTACHVGGGTWTCTA-3′) primers. The amplified PCR fragments were purified using Agencourt AMPure XP beads in order to generate a sequencing library. The sequencing was conducted on an Illumina MiSeq platform using the paired-end (PE) method with 300 bp read length and 40% phiX quality control. The sequencing data have been deposited in the GenBank short-read archive (SRA) with accession number: PRJNA720241.

### Raw data process pipeline

Trimmomatic^19^ v0.39 was used to filter low-quality reads with the parameters “TRAILING:20,” “SLIDINGWINDOW:50:20,” and “MINLEN:50.” Following quality control, FLASH^20^ v1.2.9 was employed to merge paired reads with a minimum overlap length of 10 bp and an allowable mismatch density of 0.2 in the overlapping region. Subsequently, USEARCH^21^ v11.0.667 was used to cluster aligned reads with UPARSE at a similarity threshold of 0.97. For taxonomy assignment of OUT representative sequences, QIIME^22^ v1.9 was employed via RDP method with reference database SILVA 128^23^ and bootstrap value set at 0.7 for enhanced accuracy and reliability of results obtained from this analysis approach. The resulting table contained information on both OUT abundance as well as taxonomic classification data.

### Microbial diversity analysis and statistic comparisons

Alpha diversity was calculated using Mothur^24^ v1.4 on rarified OUT tables. The Shannon indices of community biodiversity were assessed based on the abundance of OTUs. Principal coordinates analysis was performed using MicrobiomeAnalyst^25^.

### Statistical analysis

The data acquired in this study were subjected to analysis utilizing the software package GraphPad Prism 8. The data are presented as the means ± SEM, accompanied by the number of samples (*n*) in each group. After histogram and Kolmogorov–Smirnov analysis, the obtained data accorded with normal distribution. For comparison between the two groups, an unpaired *t*-test was used to analyze the statistical significance. For comparisons among more than two groups, one-way analysis of variance (ANOVA), followed by Tukey’s *t*-test was used. Results with a *p*-value less than 0.05 were deemed statistically significant.

## Results

### Body weight and daily alcohol comsumption

**Figure 1:**
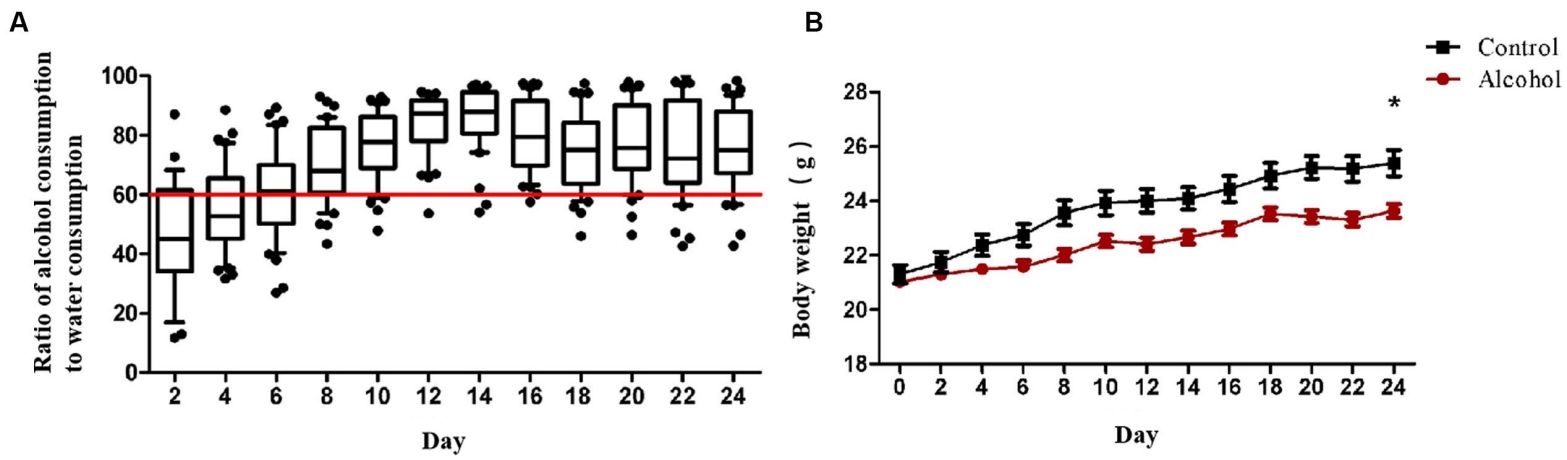
**(A)** The radio of alcohol solution intake to water. The concentration of alcohol solution was 3, 6, and 10% (v/v), and the concentration gradient of alcohol was increased every 4 days to train the alcohol adaptation ability of mice. After 8 days, the alcohol solution concentration was increased to 10% and feeding was continued for another 16 days. Each data point represents the ratio of alcohol consumption to water consumption in that cage (*n* = 8). **(B)** Body weight of the two groups during 24 days feeding. Control group (*n* = 5), alcohol group (*n* = 25) ^*^p < 0.05.

In addition to the withdrawal symptoms after alcohol withdrawal, alcohol addiction patients also have clinical signs such as gradually increasing alcohol consumption and strong alcohol addiction. After establishing the alcohol addiction model of C57BL/6 mice with double bottle supply of water solution and alcohol solution, the body weight and alcohol preference ratio of mice in the alcohol group and the control group were compared. The alcohol preference ratio was calculated as the ratio (percentage) of the weight of alcohol solution consumed by the mice to the total weight of water and alcohol solution consumed at the same time, and this result was used to compare the degree of alcohol preference of the mice. It can be seen from Figure 1A that from day 0 to day 12, when the concentration of alcohol solution increased from 3% to 6% and then to 10%, the intake of alcohol solution of mice gradually increased, and the alcohol preference ratio increased from 53% to 68% and then to 88%. After day 12, the alcohol solution concentration was maintained at 10%, and the alcohol preference ratio of the drinking mice as a whole was also maintained at 70% and above. During the drinking period, the weight change of mice was shown in Figure 1B. The weight gain of mice in the alcohol drinking group was 6.8% lower than that in the control group. On the 24th day of drinking, the weight change of mice in the two groups was significantly different (*p* < 0.05).

### Mice withdrawal performed anxiety and depression

**Figure 2:**
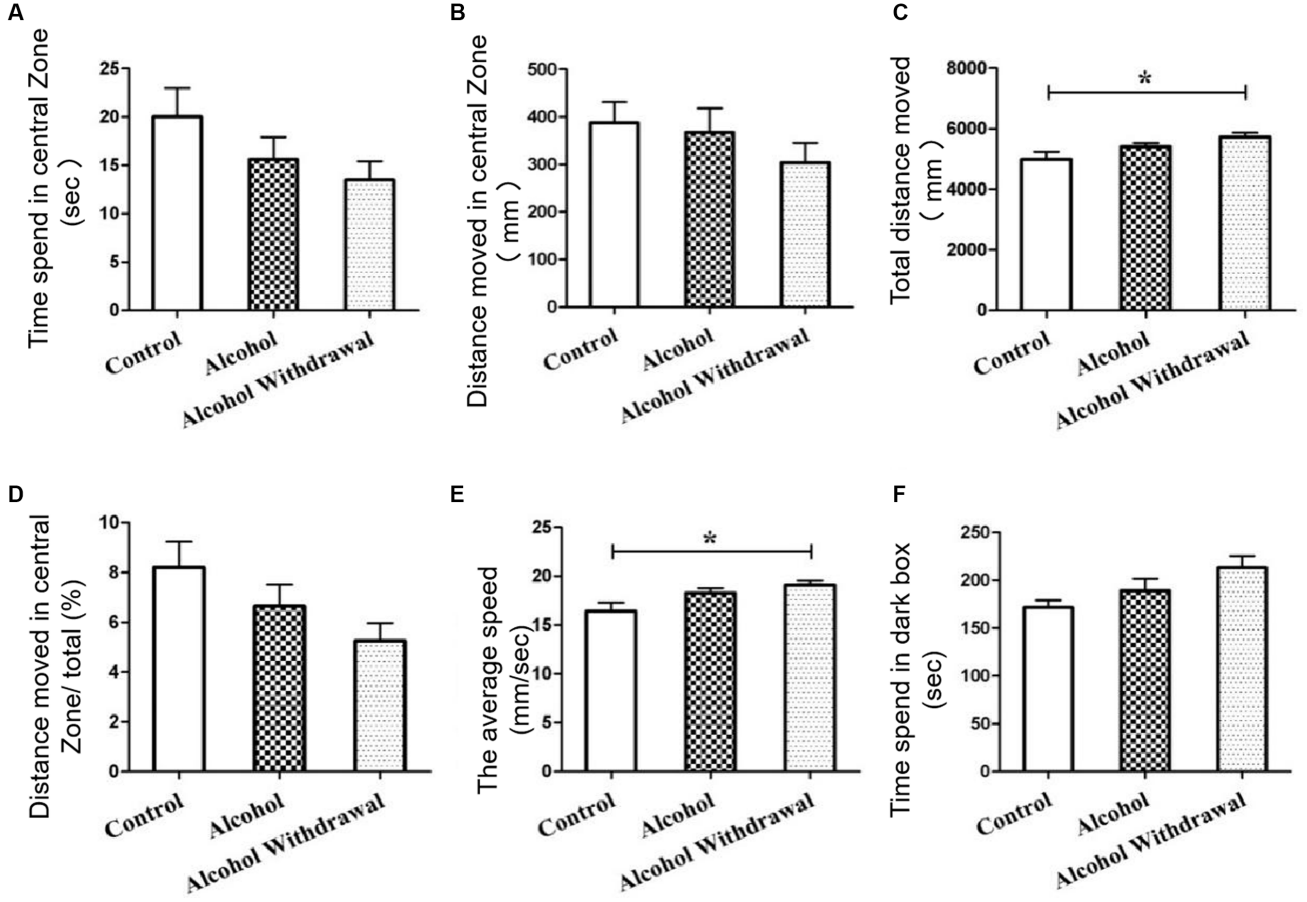
Behavioral tests. **(A)** Time spent in central zone. **(B)** Distance moved in central zone. **(C)** Total distance moved. **(D)** Distance moved in central zone/total (%). **(E)** The average speed. **(F)** Time speed in dark box. Control group (*n* = 5), alcohol group (*n* = 25), alcohol withdrawal group (*n* = 25).

OFT results showed that the mean movement time of alcohol withdrawal group (13.5 s) was shorter than that of alcohol group (15.6 s) and control group (20.0 s) (Figure 2A). The mean distance in the central region of mice in alcohol withdrawal group (304.0 mm) was lower than that in alcohol group (367.6 mm) and control group (388.2 mm) (Figure 2B). The ratio of distance traveled in the central region to total distance in alcohol withdrawal group (5.3%) was lower than that in alcohol group (6.7%) and control group (8.2%) (Figure 2D). These three sets of data suggest that BALB/c mice develop anxious and depressed mood with reduced exploratory activity in the central region after alcohol withdrawal. However, there was no statistically significant difference compared with control and pre-withdrawal mice. The total distance and average speed of movement were used to analyze the autonomous behavior of mice in the open field box, as shown in Figures 2C,E in control group (*p* < 0.05). The voluntary behavioral activity of mice increased after abstinence. According to LDT analysis, as shown in Figure 2F, the mean retention time in the dark box of alcohol withdrawal group, alcohol group and control group was 213.4 s, 189.4 s and 171.5 s, respectively. After alcohol withdrawal, the mice spent more time in the dark box, and the mice in the drinking group spent slightly more time in the dark box than the control group. The mice in the drinking group showed a trend of anxiety and depression during drinking and the trend increased after alcohol withdrawal.

### The change of microbiota composition in the alcohol dependence group

**Figure 3:**
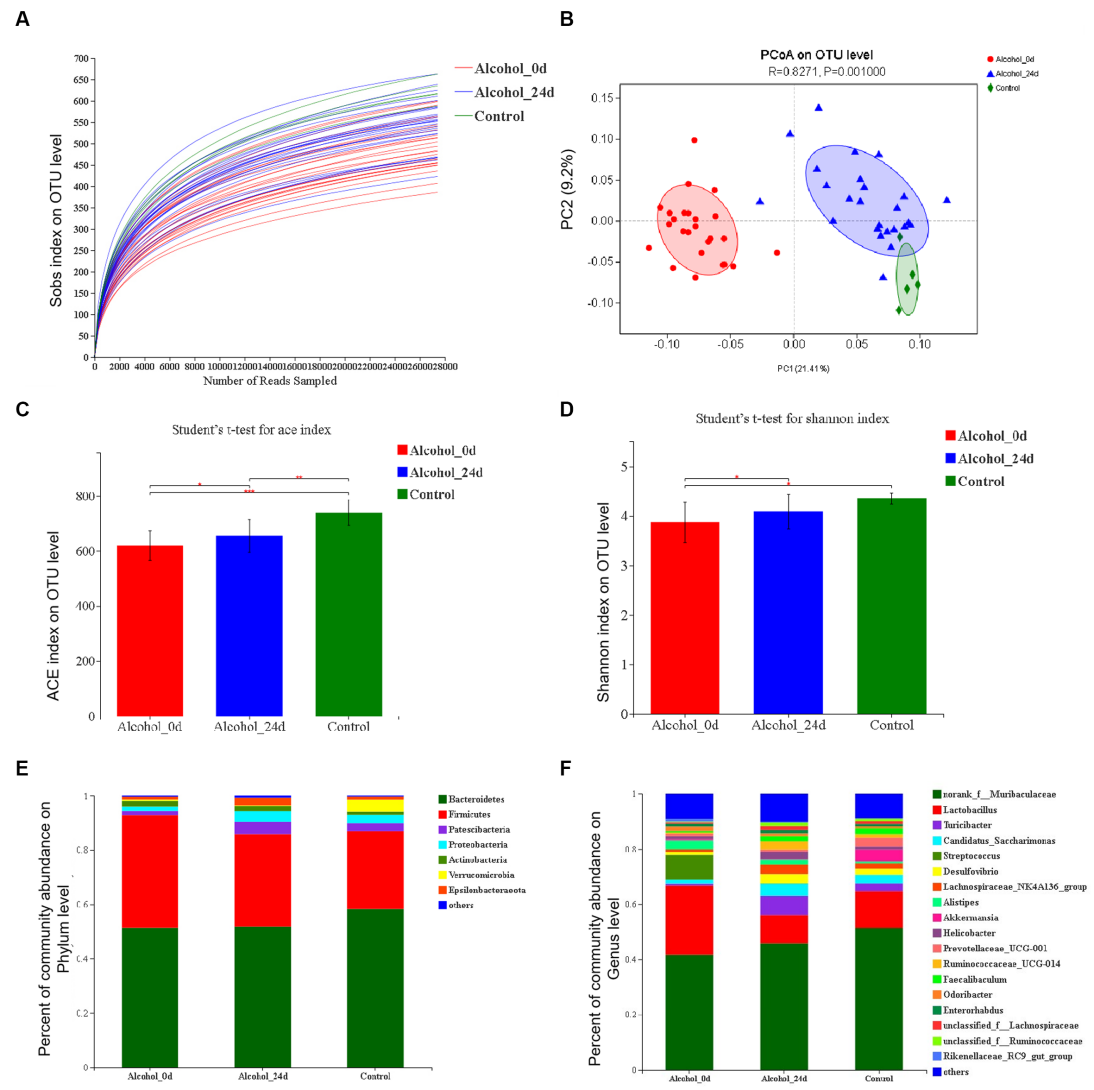
The impact of excessive alcohol intake on the composition of intestinal microbiota. **(A)** Dilution curve. The horizontal coordinate is the amount of randomly selected sequencing data, and the vertical coordinate is the observed species richness index. **(B)** Principal component analysis (PCA) of the samples on OTU level. The fecal microbiotas of the three groups exhibited distinct clustering patterns based on their community composition. **(C)** ACE diversity index. **(D)** Shannon diversity index. **(E,F)** Control group (*n* = 5), Alcohol 0d (*n* = 25), Alcohol 24d (*n* = 25). The changes of intestinal bacterial patterns at the phylum level and genus level and in mice were evaluated through 16S sequencing. The horizontal coordinate is the sample name, the vertical coordinate is the proportion of species in the sample, the different colors of the column represent different species, and the length of the column represents the relative abundance of the species.

The dilution curves of the three groups in Figure 3A tended to be flat with the increase of the number of analyzed sequences, indicating that the amount of data sequenced in this study was reasonable and had covered almost all the bacteria in the sample, and few new species will appear even if the amount of sequencing continues to increase.

The Alcohol_24d group was close to the control group and far from the Alcohol_0d group in the figure, and these three groups were able to separate on the PC1 and PC2 axes (Figure 3B). The results also indicated that the species composition and abundance of GM in mice were changed after alcohol consumption, excluding the influence of other conditions.

Figures 3C,D show a comparison of the alpha diversity indices ACE and Shannon. ACE index showed that compared with the Alcohol_0d group, the GM species richness of the Alcohol_24d and control groups was significantly increased, and the GM species richness of the control group was significantly higher than that of the Alcohol_24d group (*p* < 0.01). Species diversity assessed by Shannon index showed that compared with the Alcohol_0d group, both the Alcohol_24d and control groups had significantly increased GM species diversity, and the control group had slightly higher species diversity than the Alcohol_24d group. This suggests that alcohol consumption leads to a decrease in GM species richness and diversity in mice.

The species abundance and community composition of the samples were analyzed at different taxonomic levels. The difference of intestinal flora between the alcohol dependent mice and healthy mice was analyzed, at the phylum level (Figure 3E), the top two dominant bacteria in the three groups were *Bacteroidetes* and *Firmicutes*, and their proportions in the three groups were different. In order of relative abundance of species, *Actinobacteria* and *Proteobacteria* were next in Alcohol_0d group, and *Patescibacteria* and *Proteobacteria* were the third and fourth in Alcohol_24d group and control group. At the genus level (Figure 3F), the first two dominant bacteria in the three groups were norank_f_*Muribaculaceae* and *Lactobacillus*, and the abundance of *Lactobacillus* decreased in Alcohol_24d group. According to the relative abundance of species, *Streptococcus* and *Saccharimonas* had a higher proportion in Alcohol_0d group, and *Turicibacter* and *Candidatus_saccharimonas* had an increased proportion in Alcohol_24d group. The *Turicibacter* and *Prevotellaceae*_UCG_001 genera accounted for a higher proportion in the control group. The community composition and species richness of Alcohol_24d group changed compared with Alcohol_0d group and control group.

### Transplantation of faecal microbiota from health mice restore the composition of alcohol dependence mice gut microbiota

**Figure 4:**
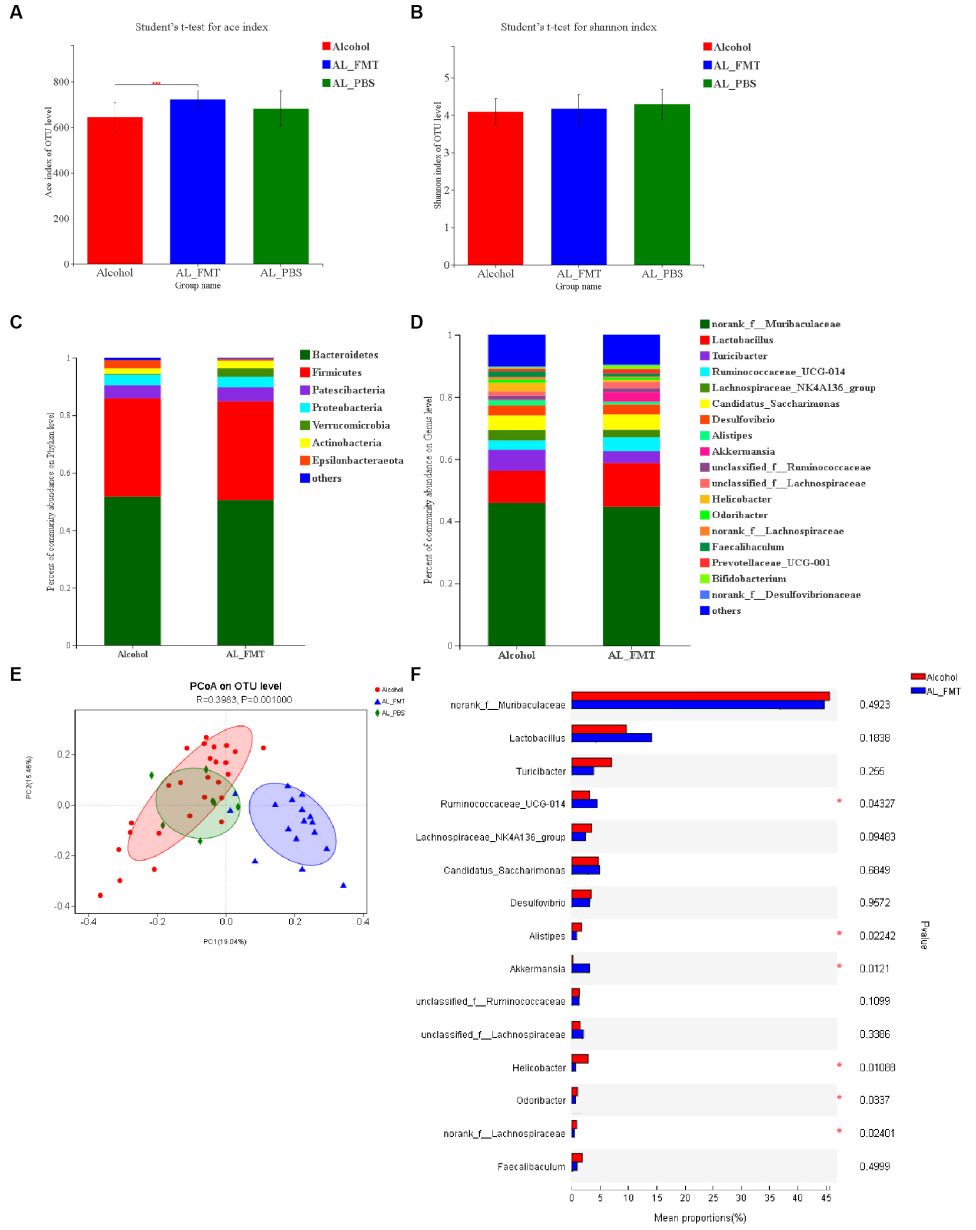
Reshaping effect of fecal bacteria transplantation on intestinal flora in alcohol-dependent mice. **(A)** ACE diversity index. **(B)** Shannon diversity index. **(C,D)** The changes of intestinal bacterial patterns at the phylum level and genus level and in mice were evaluated through 16S sequencing. Alcohol group (*n* = 25), AL_FMT group (*n* = 17) and AL_PBS group (*n* = 6). The horizontal coordinate is the sample name, the vertical coordinate is the proportion of species in the sample, the different colors of the column represent different species, and the length of the column represents the relative abundance of the species. **(E)** Principal component analysis (PCA) of the samples on OTU level. **(F)** Student *t*-test for significance of different species at genus level. ^*^p < 0.05.

ACE index evaluated species richness, and the richness of AL_FMT group was higher than that of AL_PBS group. Compared with alcohol group, GM species richness of mice in AL_FMT group was significantly increased (*p* < 0.001) (Figure 4A). Shannon index showed that GM species diversity of mice in alcohol group, AL_FMT group and AL_PBS group had no significant difference (Figure 4B). These results indicated that FMT to drinking mice would increase the GM species richness of mice, but there was no significant change in species diversity. The difference of intestinal flora between the AL_FMT mice and healthy mice was analyzed, at the phylum level (Figure 4C), the abundance of *Verrucomicrobia* increased while the abundance of *Epsilonbacteraeota* decreased in AL_FMT mice. At the genus level (Figures 4D,F), the contents of two genera (*Ruminococcaceae* UCG-014 and *Akkermansia*) in AL_FMT group was significantly increased, while the abundance of *Helicobacter*, *Odoribacter*, *Lachnospiraceae* and *Alistipes* was significantly decreased in AL_FMT group. The species composition of AL_FMT and alcohol group could be separated clearly by PCA, while the AL_PBS group was similar to alcohol group (Figure 4E). It indicated that FMT could alter the GM composition and abundance in alcohol dependent mice.

### FMT ameliorates alcohol-induced behavioral deficits

**Figure 5:**
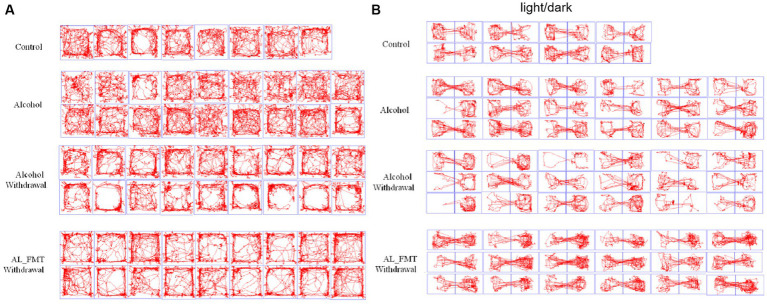
**(A)** Representative animal track in OFT. The central zone is represented by the blue square area. **(B)** Representative animal track in LDT.

After 14 days of FMT, alcohol-dependent mice were subjected to behavioral tests before and after alcohol withdrawal. Behavioral tests were performed on alcohol-addicted mice receiving FMT from healthy donors (AL_FMT group), and behavioral tests were performed on AL_FMT mice after 24 h of alcohol withdrawal (AL_FMT_Withdrawal group). Behavioral tests were performed in the AL_PBS group. Behavioral tests were performed in the AL_PBS group 24 h after alcohol withdrawal (AL_PBS_Withdrawal group). The motion trajectories of OFT and LDT are shown in Figures 5A,B, compared with the control group, the alcohol group mice preferred to move around the open field and in the dark box after alcohol withdrawal 24 h, depression-like behavior was improved when they received the microbiome from control mice. There was no significant difference in mice behavior between the alcohol group and the control group without alcohol withdrawal.

### FMT increased the content of 5-HT in the intestinal and brain tissues of alcohol-dependent mice, and promoted the expression of 5-HT receptor

**Figure 6:**
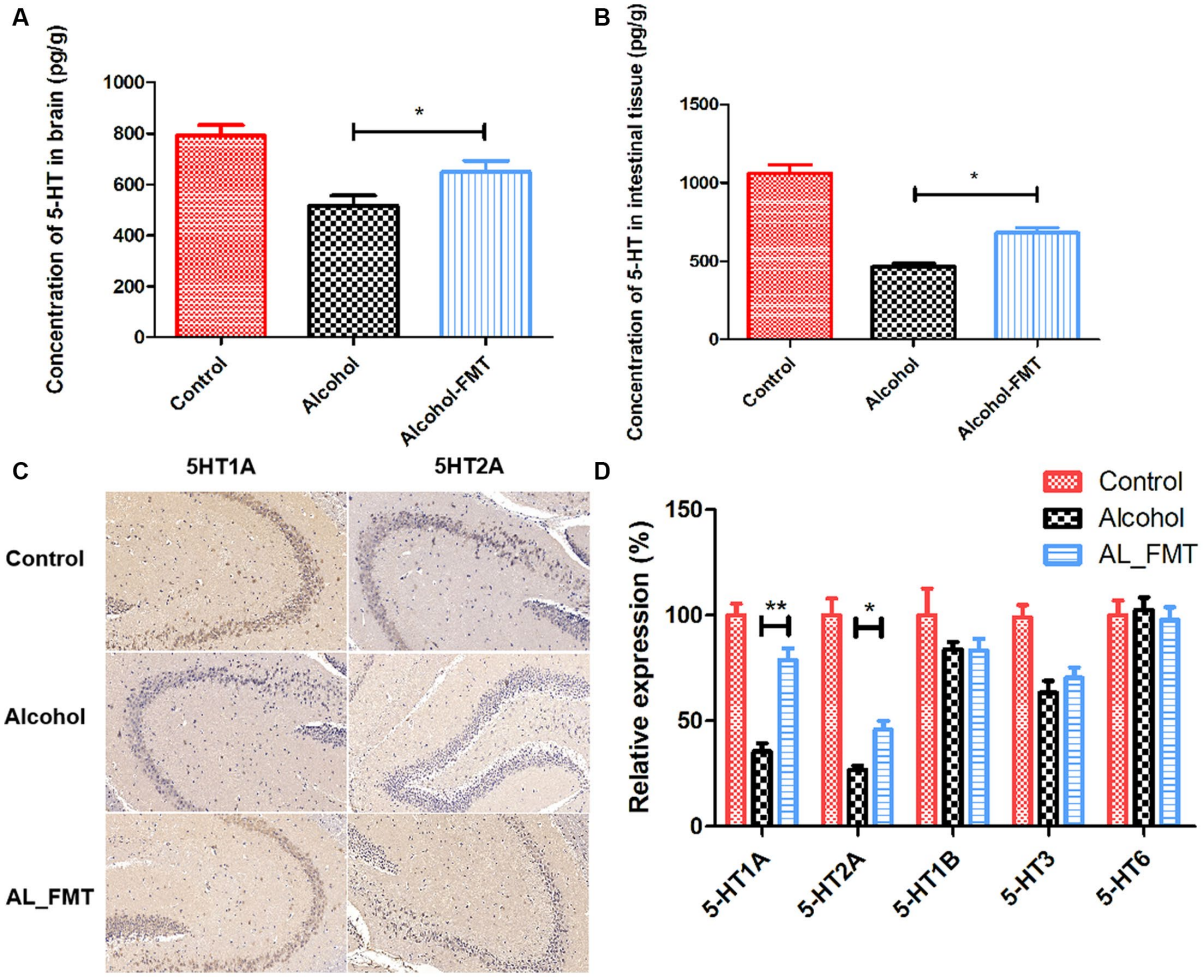
**(A)** The concentration of 5-HT in brain. **(B)** The concentration of 5-HT in intestinal tissue. **(C)** 5-HT1A and 5-HT2A were stained by immunohistochemistry on hippocampus sections from each group. Scale bar = 50 μm. **(D)** Quantification of genes related to 5-HT receptors associated with substance dependence. Control group (*n* = 5), alcohol group (*n* = 25) and AL_FMT group (*n* = 17). Expression levels of 5-HT1A, 5-HT2A, 5-HT1B, 5-HT3 and 5-HT6 in different groups of mouse hippocampus were measured by qRT-PCR. The acquired cycle threshold (CT) was normalized to the CT of the β-actin. Data are presented as the mean ± SD of three independent experiments, with each experiment being comprised of four individual measurements. Unpaired *t*-tests were performed for significance. ^*^p < 0.05 and ^**^p < 0.01.

As a major neurotransmitter, 5-hydroxytryptamine (5-HT) can regulate the secretion of opioid peptide, gamma-aminobutyric acid, glutamic acid and other transmitters, and plays a key regulatory role in many aspects of emotion, cognition, substance dependence and so on. What’s more notable is that 95% of 5-HT is produced in the enteric nervous system. Therefore, we explored the effect of FMT on 5-HT content, the results showed that the 5-HT content was significantly increased in alcohol dependence mice intestinal and brain tissues after receiving the fecal microbiome from healthy mice (Figures 6A,B). Considering that the abnormal function of some 5-HT receptors is also a cause of substance dependence, we examined a range of receptors (5-HT1A, 5-HT2A, 5-HT1B, 5-HT3, 5-HT6) associated with it. As shown in Figure 6D, the expression of 5-HT1A and 5-HT2A receptors was decreased in alcohol-dependent mice, while FMT significantly increased the mRNA expression level of 5-HT1A and 5-HT2A. We further examined the presence of 5-HT receptors in the hippocampus by immunohistochemical assay, the results were consistent with those of qPCR (Figure 6C). It indicated that FMT may alleviate alcohol withdrawal syndrome possibly through increasing the 5-HT and its receptor 5-HT1A and 5-HT2A.

### FMT promote the expression of tight junction protein ZO-1 and occludin

**Figure 7:**
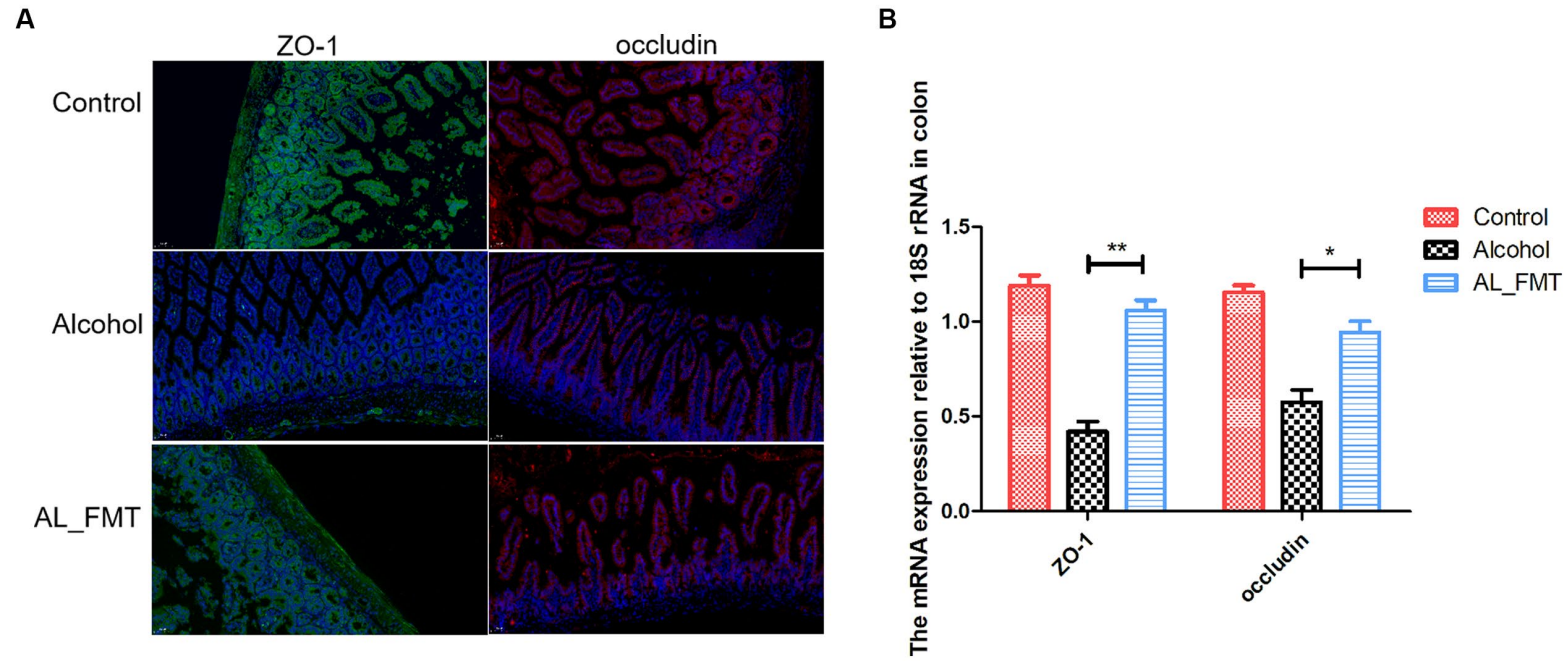
**(A)** Immunofluorescence detection of tight junctions related proteins ZO-1 (green fluorescence) and occludin (red fluorescence) in colon tissues. Nuclei were counterstained with DAPI, emitting blue fluorescence. Scale bar = 50 μm. **(B)** The expression levels of ZO-1 and occludin were measured by qRT-PCR. Control group (*n* = 5), alcohol group (*n* = 25) and AL_FMT group (*n* = 17). ^*^p < 0.05 and ^**^p < 0.01.

Alcohol metabolites can lead to the destruction of tight junction proteins in the intestine. The results of fluorescence quantitative PCR showed that, compared with healthy mice, the mRNA transcription levels of ZO-1 and occludin in the intestine of alcohol-dependent mice decreased by 3–4 times, respectively, and after FMT treatment, ZO-1 and occludin transcription levels recovered to healthy levels (Figure 7B). To further confirm the structural integrity of TJs, the immunofluorescence technique was used to detect the protein expression level of ZO-1 and occludin in colon. The results showed that the expression level of ZO-1 and occludin decreased significantly in alcohol-dependent mice, while FMT was able to ameliorate the changes of the two proteins (Figure 7A).

### FMT reduces the increased of LPS and FABP2 levels in the alcohol dependence mice serum

**Figure 8:**
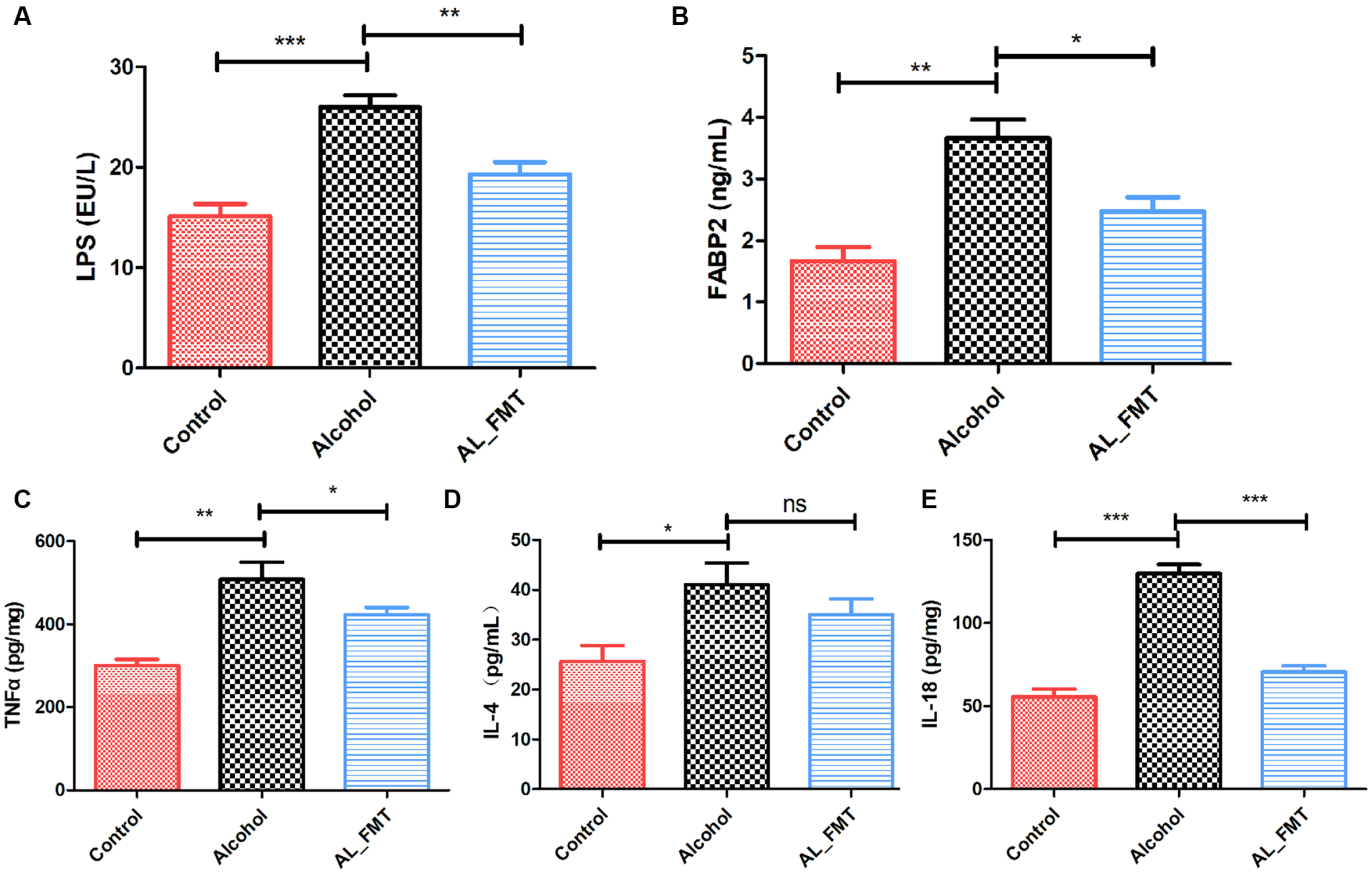
Effects of FMT on alcohol-induced inflammatory reaction. **(A,B)** The serum levels of LPS and FABP2. **(C–E)** The concentrations of pro-inflammatory cytokines in the spleen. Control group (*n* = 5), alcohol group (*n* = 25) and AL_FMT group (*n* = 17). ^*^p < 0.05 and ^**^p < 0.01.

In order to ascertain the impact of FMT on intestinal permeability, the ELISA analysis was performed to determine the serum levels of LPS and FABP2, which served as biomarkers of intestinal barrier permeability, as shown in Figures 8A,B. Compared to the control group, the LPS and FABP2 in alcohol dependence mice serum increased, while FMT significantly decreased the change levels, suggesting the reparative impact of FMT on intestinal leakage.

### FMT attenuates alcohol-induced cytokines release in the spleen

To further investigate the influence of FMT on the pro-inflammatory factor, a series of ELISA assays were performed. The results revealed that FMT significantly decreased the levels of TNF-α, IL-4 and IL-18 (Figures 8C–E), which indicated that FMT could ameliorate the inflammatory reaction in alcohol dependence mice.

## Discussion

Alcohol is an addictive substance. Long-term drinking can lead to psychological and physiological dependence on alcohol, forming alcohol dependence. Patients with alcohol dependence have alcohol withdrawal symptoms such as depression, anxiety and other brain disease (Peacock et al., 2018). Currently, several drugs are used for the alcohol treatment, but these drugs are less effective and have some side effect (Fernandez et al., 2015). Thus, it’s necessary to find new interventions for alcohol dependence treatment. The microbial-gut-brain axis is a two-way communication pathway that includes the central nervous system, neuroendocrine and neuroimmune systems. Through reconstructing gut microbes to ameliorate neurological diseases is considered a promising area of research (Cryan et al., 2020). In this study, the effect of FMT on alcohol dependence mice was investigated. Our results indicated that FMT ameliorate alcohol dependence symptoms by reducing the inflammation and increasing the production of 5-HT in the brain gut axis.

Generally, research on gut microbiome involvement in nervous system disease has been the focus of people’s attention (Erny et al., 2021; Fulton et al., 2021), however, only a small part of publications is about drug dependence, especially alcohol dependence (Angoa-Perez and Kuhn, 2021). In this study, the alcohol dependent mice model was constructed by feeding with two-bottle drinking mode to simulate free drinking. After a period of training, alcohol-dependent mice showed a significant preference for alcohol and showed depression-liked behavior, which indicated that the alcohol dependent model had been successfully established. Moreover, transferring the gut microbiota of healthy mice to alcohol dependent mice could alleviate the depression-like behavior and the dependence on alcohol. In order to understand the mechanism of FMT, we conducted a follow-up investigation.

The formation mechanism of alcohol dependence is complex, among which depression is one of the causes of addiction (Leclercq et al., 2014). As a major neurotransmitter, 5-hydroxytryptamine (5-HT) can regulate the secretion of opioid peptide, gamma-aminobutyric acid, glutamic acid and other transmitters, and plays a key regulatory role in many aspects of emotion, cognition, substance dependence and so on (Bellono et al., 2017). There is a negative correlation between 5-HT and drinking behavior. In the experiments of alcohol-dependent mice, monkeys and other animals, adding the precursor substances synthesized by 5-HT into the feed can reduce the drinking behavior of animals (Agirman et al., 2021). The abnormal function of 5-HT and its receptor has an important relationship with the occurrence and development of mental disorders [such as substance addiction and depression) (Chen et al., 2022]. Thus, we measured the content of 5-HT in the brain, the results showed that the concentration of 5-HT in alcohol dependent mice brain decreased 30% compared with that of control mice.

More than 95% of body’s 5-HT was produced by the enteric nervous system (ENS) and some species of bacteria, such as *Enterococcus*, produce 5-HT (Clarke et al., 2014). What’s more, tryptophan is an essential amino acid precursor to 5-HT. Intestinal flora decompose tryptophan in food into a series of small molecular metabolites with neural activity, including: Formation of 5-HT through tryptophan hydroxylase; Formation of TRY or generation of IAA and ILA through tryptophan decarboxylate, which is related to maintaining mucosal integrity and permeability; Formation of kynurenic acid, which is related to the nervous system inflammation (Kennedy et al., 2017). Hence, we investigated the effect of reconstructing intestinal flora on alcohol dependence and the expression of 5-HT through FMT. The results showed that FMT alleviated the alcohol dependent mice anxiety-like and alcohol-seeking behavior. Meanwhile, the content of 5-HT in alcohol dependent mice brain was significantly increased after FMT. Additionally, accumulating evidence indicates that the decreased expression of 5-HT receptor in hippocampus is related to the cognitive loss and depression by alcohol consumption (Brunetti et al., 2012; Bhattarai et al., 2017; Borroto-Escuela et al., 2017). Thus, we determined the receptor of 5-HT in hippocampal region via qRT-PCR and immunochemistry. The results showed that FMT improved the expression of 5-HT1A and 5-HT2A in hippocampus. It indicated that FMT may alleviate the alcohol-induced depression by increasing the 5-HT content and its receptor 5-HT1A and 5-HT2A in the hippocampus.

As a result, cells produce a large number of inflammatory cytokines (IL-6, IL-8, IL-10, TNF-α), activate microglia cells, and cause brain nerve damage, which is associated with the symptoms of alcohol craving. Similarly, LPS and a series of inflammatory cytokines in our alcohol dependent mice was significantly increased in our study.

Alcohol intake can lead to intestinal flora imbalance and damage intestinal tightness, resulting in increased intestinal permeability and “intestinal leakage,” in which bacterial products such as lipopolysaccharide (LPS) reach the liver through the intestinal mucosa through the blood circulation, stimulating the activation of TLR4 and activating NLRP3 inflammatories (Jadhav et al., 2018). In our study, we examined the impacts of FMT on the crucial proteins of tight junctions. Immunofluorescence histochemistry and qRT-PCR showed that the FMT up-regulated the decreased the expression levels of ZO-1 and occludin caused by alcohol exposure. Furthermore, FABP2, the molecule of biomarker for intestinal epithelium paracellular integrity, was also up-regulated by FMT. Generally, the intestinal flora imbalance and damage intestinal tightness, resulting in increased intestinal permeability and “intestinal leakage,” in which bacterial products such as lipopolysaccharide (LPS) reach the liver through the intestinal mucosa through the blood circulation, stimulating the activation of TLR4 and activating NLRP3 inflammatories. The subsequent inflammatory response contributes to the depression caused by alcohol (Leclercq et al., 2012). Considering our results showed FMT has a repairing effect on intestinal barrier, we further determined the LPS in serum. The level of LPS in alcohol dependent mice was higher than the control mice as expected. In contrast, the FMT reversed this change, possibly due to the improvements in intestinal leakiness. Additionally, several studies have shown that the spleen’s inflammatory response affects brain and gut function, indicating its importance in the brain-gut axis (Saand et al., 2019; Zhang et al., 2021). Consequently, we measured splenic levels of pro-inflammatory cytokines in different groups. As expected, FMT significantly reduced the expression level of TNF-α, IL-4 and IL-18. Combined, FMT alleviated brain gut dysfunction in alcohol dependent mice by reducing inflammation.

In order to test whether FMT modulated gut microbiota following microbial therapy, 16S rDNA (V3 + V4) gene sequencing was performed on the different groups of mice to analyze bacterial taxonomic composition. At the phylum level, the abundance of *Verrucomicrobia* decreased while the abundance of *Epsilonbacteraeota* increased in alcohol mice compared with control mice. After fecal microbiota transplantation, abundance of *Verrucomicrobia* and *Epsilonbacteraeota* was close to the control group. At the genus level, the contents of two genera (*Ruminococcaceae* UCG-014 and *Akkermansia*) in AL_FMT group was significantly increased, while the abundance of *Helicobacter*, *Odoribacter*, *Lachnospiraceae* and *Alistipes* was significantly decreased in AL_FMT group. UCG-014 belongs to the rumen bacteria family, and its abundance variation is associated with neurological diseases (Pizarro et al., 2021). A human study further confirmed that UCG-014 is positively correlated with 5-HT content in patients with alcohol consumption (Hao et al., 2023). *Akkermansia* is considered to be a potentially beneficial bacterium that not only enhances intestinal barrier function, but also ameliorates depressive disorders in murine by up-regulating 5-HT in hippocampus (Guo et al., 2022). *Lachnospiraceae* belong to the family Trichomonas. It has been found that the decreased abundance of *Spirillaceae* may be related to cognitive impairment, and it is significantly enriched in substance dependent patients. The abundance of *Alistipes* was positively correlated in substance dependence patients (Vincent et al., 2016). However, the specific mechanism of action of the microflora in alcohol dependence needs further study. FMT may improve the anxiety-like behavior and alcohol-seeking behavior of dependent mice by reversing the abundance of potentially beneficial bacteria *Verrucobacteria*, *Lactobacillus*, *Ruminococcaceae*_UCG-014 and *Akkermansia*, reducing the abundance of potentially harmful bacteria.

In conclusion, this study reveals that FMT treatment alleviates the anxiety-like behavior and alcohol-seeking behavior in dependent mice alcohol dependent. Additionally, FMT treatment enhanced intestinal barrier integrity and changed the composition of gut flora. Furthermore, FMT ameliorated the inflammatory reaction and increased the expression of 5-HT and its receptor (5-HT1A and 5-HT2A) in alcohol dependence mice. Our results demonstrated that reconstructing the gut microbiota by FMT may alleviate alcohol withdrawal syndrome possibly through reducing the inflammatory response and regulating the 5-HT system in the brain of mouse, support that FMT might be a promising intervention strategy for alcohol dependence treatment, although more data about safety and clinical trials are needed in humans.

## Data availability statement

The datasets presented in this study can be found in online repositories. The names of the repository/repositories and accession number(s) can be found in the article/supplementary material.

## Ethics statement

The animal study was approved by the Ethics Committee of China and conducted in accordance with ethical standards. The study was conducted in accordance with the local legislation and institutional requirements.

## Author contributions

QL conceived and directed the study. DL carried out the experimental operation and wrote the manuscript. WL analyzed the data. ZH and WZ gave experimental help. HL provided valuable suggestions of the manuscript. All authors contributed to the article and approved the submitted version.
